# Soy, Rice and Oat Drinks: Investigating Chemical and Biological Safety in Plant-Based Milk Alternatives

**DOI:** 10.3390/nu15102258

**Published:** 2023-05-10

**Authors:** Roberta Giugliano, Noemi Musolino, Valentina Ciccotelli, Carla Ferraris, Valentina Savio, Barbara Vivaldi, Carlo Ercolini, Daniela Manila Bianchi, Lucia Decastelli

**Affiliations:** 1 National Reference Laboratory of Pesticides in Cereals and Feed (NRL), Istituto Zooprofilattico Sperimentale del Piemonte, Liguria E Valle D’Aosta, Piazza Borgo Pila 39/24, 16129 Genoa, Italy; 2 National Reference Centre for the Detection of Substances and Products Causing Allergies or Intolerances in Food (CReNaRiA), Istituto Zooprofilattico Sperimentale del Piemonte, Liguria e Valle D’Aosta, Via Bologna 148, 10154 Turin, Italy

**Keywords:** plant-based milk alternatives, allergies, lactose, chemical safety, microbiological safety, pesticides residues

## Abstract

During the last decades, plant-based milk has become very appreciated by consumers, becoming a staple ingredient, especially for alternative breakfasts. Milk contains lactose, which is a sugar hydrolysed by the lactase enzyme. Lactose intolerance and lactose malabsorption are very common food intolerances among individuals. However, a lot of consumers consider themselves as lactose intolerant on the basis of self-reported intolerance and start to avoid dairy products, ignoring that plant-based milk alternatives are not nutritionally comparable to animal milk, especially in terms of protein intake. The aim of this study is to grow folder knowledge of the security of plant-based drinks, helping competent authorities to issue a risk assessment and to apply national plans about consumer safety. Results show that proper sanitary practices, such as pasteurization, are necessary in plant-based milk alternatives as well as in dairy milk. Chemical analysis has highlighted that there are no pesticide risks for consumers.

## 1. Introduction

Over the past decade, an increase in demand for alternative healthy foods from consumers has been recorded. Recent studies estimate that the global milk alternatives market could potentially exceed revenues of $38 billion by 2024. Asia-Pacific (APAC) is the fastest growing region in the market, while North America remains the biggest consumer region (Arizton Advisory and Intelligence 2019, website). According to IRI 2021 data, spread by Unione Italiana Food, the Italian market recorded a +9.9% increase in plant-based products, a trend confirmed also in the first semester of 2022 (Italia Fruit News 2022, website). Due to cow milk allergy, lactose intolerance, calories and hypercholesterolemia concerns, consumption of milk alternatives such as plant-based drinks has increased, especially soya drink consumption [1,2]. The trend towards changing food lifestyles (flexitarian, vegetarian and vegan lifestyles) seems to be the main driver behind it, especially in developed countries [3,4].

Lactose is the sugar component of milk, synthesized by D-galactose and D-glucose subunits via β-1,4 glycosidic bond and hydrolysed by the enzyme lactase phlorizin hydrolase (colloquially, lactase) [5]. Lactose intolerance is one of the most common food intolerances among individuals [6], and it is defined as “the onset of gastrointestinal symptoms (i.e., diarrhoea, abdominal cramping, audible bowel, flatulence, vomiting) following a single-dose challenge of ingested lactose by an individual with lactose maldigestion, which are not observed when the person ingests an indistinguishable placebo” [7]. Lactose maldigestion refers to the non-digestion and/or non-absorption of lactose in the small intestine, and the most common cause is reduction in lactase production (also known as lactase non-persistence) during adulthood [8].

Lactose intolerance is very common among people: about 57% of people worldwide are affected by confirmed lactose intolerance [9], but the percentage may be different both among ethnic populations [9,10] and because self-diagnosis of milk intolerance is common [6]. According to the EFSA Panel on Dietetic Products, Nutrition and Allergies (NDA) [10], lactose intolerance affects about 70% of the world adult population, and in Europe, according to Lember [11], it varies from around 2% in Scandinavia to about 70% in South Italy (Sicily). On the other hand, cow’s milk allergy (CMA) reaches about 0.50%–3.50% of individuals [12,13].

In case of lactose intolerance [14,15,16], the treatment is a diet with reduced lactose content: some studies suggest that the vast majority of subjects may tolerated up to 12 g of lactose per day with no or minor symptoms, but it is not possible to determine a single threshold of lactose owing to the great variation in individual tolerances [7]. Regarding CMA, the only solution is the exclusion of cow’s milk and its derivates, and milk can be substituted by donkey milk, for example [17,18]. In addition, thanks to their growing popularity [19], plant-based beverages are also widely used to replace milk, as they are similar in sensorial aspects regarding colour, texture and sometimes flavour [20]. However, the consumption of plant-based beverages should be carried out with caution and after a proper diagnosis: in fact, according to the American Academy of Pediatrics (AAP), 10 to 14% of the infants with CMA will also become allergic to soy [21,22]. Specifically, soy-, oat- and rice-based beverages are not suitable alternatives for food protein-induced enterocolitis syndrome (FPIES) patients, as such food are common triggers [23,24].

In case of symptoms of lactose intolerance, a diagnosis can be performed through several methods. The most frequently used is HBT, based on the fermentation of undigested lactose and the consequent production of gas by intestinal microbiota, thus leading to the diagnosis of lactose malabsorption [9]. During HBT, fasted subjects drink a lactose-loaded solution (25–50 g of lactose [25]) and then breath samples are analysed to collect the value of hydrogen [13].

However, a lot of people consider themselves as lactose intolerant without any diagnostic test performed [26] and so start to avoid dairy products on the basis of self-reported intolerance. Indeed, according to Casellas et al. [27], subjects usually associate ingestion of lactose dairy products with symptoms of lactose intolerance but without diagnosis of insufficient lactase activity.

Looking carefully, commercial plant-based beverages are not nutritionally comparable to animal milk, as most consumers think. Milk and dairy products are an important source of proteins (i.e., caseins and whey proteins), essential amino acids, fats [28] and micronutrients such as calcium, vitamin D, potassium, magnesium and riboflavin [6]. The nutritional composition of plant-based beverages is different according to the raw materials used to produce them: for example, oat milk is a source of bioactive compounds such as β-glucan and soy milk contains isoflavones and phytosterols. In particular, the protein content of plant-based drinks can be lower than we would expect, and this is because the principal ingredient is water [19,29]. Approximately 50% of commercial plant-based milk alternatives contain little (< 0.5%) or even no protein, while only selected soy-based drink analogues reach the higher protein level of dairy milk (3.7%) [29].

According to Vernia [3,19,30], these subjects’ health is more compromised and they have higher risk of developing osteoporosis because of the lack of important nutrients such as calcium provided by dairy products [19,20]. These results are in accordance with the studies of McCarthy et al. [31], Vainio et al. [32] and Schyver and Smith [33], where consumers are reported to perceive plant-based drinks as a healthier alternative to cow’s milk.

Plant-based foods and vegan diets can play a role in the composition of the gut microbiome, with beneficial effects such as reduction of inflammation, energy balance and insulin sensitivity [34,35]. Recent studies have reported that oat phenolic compounds and oat β-glucan promote weight loss and reduction of lipids in the blood thanks to the stimulation of liver function and, in the intestine, to the increasing abundance of *Bacteroides* and reduced *Firmicutes* [36]. However, as reported by other authors, there are few studies that compare the nutritional composition of different milk substitutes to guide the population about the best alternative to compose their diet of [19,36]. Attached here is Table 1, showing the nutritional values of the three types of plant-based beverages investigated and the whole cow’s milk (data were purchased by the official website of the United States government of the Department of Agriculture).

The values in Table 1 are derived through state-of-the-art chemical analyses, computations and other approaches, as reported in this link: https://fdc.nal.usda.gov/data-documentation.html (accessed on 7 May 2023). Therefore, Table 1 is calculated from results obtained by analysing commercial plant-based drinks sold in markets. As can be seen from Table 1 and by Sakkas’s team study [34], there is a wide variety of vegetable drinks in the shops that are enriched with vitamins and minerals and that achieve better nutritional values than the non-fortified ones [34].

Industrial production of plant-based drinks is basically prepared with grain milling and water addition. These two ingredients produce a slurry preparation, which is hydrolysed with enzymes and finally filtrated [37].

In terms of environmental impact, a more plant-based diet in drinks consumption will certainly support a more sustainable lifestyle [3], but microbiological and chemical safety is still to be evaluated carefully.

Microbiological studies confirm that plant-based beverages might be a good growing ground for pathogens such as *Listeria monocytogenes* and *Salmonella* spp. [38,39,40]. In addition, pesticides are widely used in cereals and legumes agriculture, and EU national authorities monitor food chain samples constantly.

In the current literature, there are few studies that take into account plant-based drink issues, and the creation of a risk database is still ongoing. The aim of this study is to increase knowledge about the security of plant-based beverages and help competent authorities to fill out risk assessment and national consumer safety plans [38].

## 2. Materials and Methods

### 2.1. Selection and Collection of Samples

In order to evaluate plant-based beverages’ chemical and microbiological safeties, the research was conducted on a representative selection of beverages available on the local markets of metropolitan Turin area (North Italy). Total samples consisted of 33 soy drinks, 10 oat drinks and 17 rice drinks. Each category included different volumes, brands, lot numbers, product lines (i.e., no added sugars, organic production, flavour) and thermal processes (Table 2). Although the variety of vegetable drinks is very wide, as reported by [34], we have focused this study on the most widespread varieties on the market: soy, rice and oats [4,41].

All microbiological and chemical analyses were performed by accredited laboratories in accordance with ISO17025.

### 2.2. Pesticides Analysis

All pesticide standards, reagents and solvents were purchased from Merck (Darmstadt, Germany). Below are listed the pesticides detected: 2-Pheylphenol, Acrinathrin, Aldrin, Azoxystrobin, Bifenthrin, Bixafen, Boscalid, Bromopropylate, Bromuconazole, Cadusafos, cis-Chlordane, trans-Chlordane, Chlorfenvinphos, Chlorpropham, Chlorpyrifos, Chlorpyrifos-methyl, Cyfluthrin, λ-Cyhalothrin, Cypermethrin, Cyprodinil, p,p’-DDD e o,p’-DDT, p,p’-DDE, p,p’-DDT, cis-Deltamethrin, Diazinon, Dieldrin, Difenoconazole, α-Endosulfan, β-Endosulfan, Endosulfan-sulphate, Endrin, Endrin-ketone, EPN, Esfenvalerate, Ethion, Etofenprox, Famoxadone, Fenarimol, Fenazaquin, Fenitrothion, Fenpropathrin, Fenpropimorph, Fenthion, Fenvalerate, Fipronil, Fipronil-sulfone, Flucythrinate, Fludioxonil, Fluquinconazole, τ-Fluvalinate, α-HCH, β-HCH, Heptachlor, Heptachlor-endo-epoxide (isomer A, trans), Heptachlor-exo-epoxide (isomer B, cis), Iprodione, Isocarbophos, Isoprothiolane, Kresoxim-methyl, Lindane (γ-HCH), Malaoxon, Malathion, Mepanipyrim, Methacrifos, Metalaxyl, Methoxychlor, Metolachlor, Oxadixyl, Oxychlordane, Paraoxon-methyl, Parathion, Parathion-methyl, Pendimethalin, Permethrin, Phenthoate, Phosalone, Phosmet, Piperonyl-butoxide, Pirimiphos, Pirimiphos-methyl, Procymidone, Profenofos, Propargite, Pyridaben, Pyrimethanil, Resmethrin, Spiromesifen, Tebufenpyrad, Tefluthrin, Tetramethrin, Tolclofos-methyl, Triadimefon, Trifluralin and Vinclozolin. Triphenylphosphine (TPP) was used as internal standards.

Samples were treated using the SweEt method and injected in GC-MS/MS (Thermo SCIENTIFIC TRACE 1300 coupled with TSQ 8000 Evo) equipped with an AS 3000 autosampler. Blank reagents, blank matrices and fortified matrices were analysed in every analytical batch.

Xcalibur software was used for mass spectrometer control and data acquisition. Data analysis was performed with Trace Finder software. The GC column was a DB-5MS (30 m × 0.25 mm, 0.25 μm), the working conditions were as follows: drying gas He (purity > 99.9%) at 1.2 mL min^−1^; EI voltage 70 eV; injector temperature 250 °C; splitless mode; split flow 50 mL min^−1^; gas saver flow 10 mL min^−1^ (5 min); injection volume 1 μL. The oven temperature program was started at 50 °C for 1 min, then increased by 20 °C min^−1^ to 180 °C, increased by 5 °C min^−1^ to 270 °C and by 30 °C min^−1^ to 325 °C, then held for 2 min. The mass spectrometer operated in EI ionization in positive mode, and the MS transfer line was 260 °C. Detection was operated in SRM mode, using 2 transitions for each pesticide: 1 quantifier and 1 qualifier. In each analytical session, a post-matrix curve was prepared, using negative soy drinks. Concentrations for the matrix curves were: 0.5, 1, 2, 5 and 10 ppm. For each calibration curve, the value of the angular coefficient (R^2^ ≥ 0.97), the residues of the individual experimental points at the head and at the tail of the analytical batch (∆ ≤ 20%) and the signal-to-noise ratio (S/N ≥ 3) for the quantifier ion and for the qualifier ion of all analytes were verified.

The absence of signals attributable to analytes in the chromatograms of reagents and blank was verified to exclude the hypothesis of any contamination or matrix effects. Analyte recoveries were also verified in the fortified samples (60 ÷ 140%) in order to monitor the correct functioning of the extraction procedure.

The analytical method has been validated by NRL for Pesticides in Cereals and Feed (NRL-CF) in accordance with the SANTE/2019/12682 protocol. Validation information is reported in Tables S1 and S2 in the Supplementary Material.

### 2.3. Microbiological Assays and Organoleptic Tests

Preparations of samples and dilutions for microbiological tests were made in conformity with standard ISO 7218:2007 [42] and ISO 6887-1:2017 [43].

The study included determination of the total aerobic mesophilic bacteria count (TAMBC) (ISO 4833-1:2013) [44], enumeration of coagulase-positive staphylococci (CPS) including *Staphylococcus aureus* and other species (ISO 6888-2:2021) [45], detection and enumeration of total coliforms (ISO 4831:2006) [46], detection and enumeration of *Enterobacteriaceae* (ISO 21528-1:2017) [47], enumeration of sulphite-reducing anaerobic bacteria (SRAB) (ISO 15213:2003) [48], detection, enumeration and confirmation through cereulid-toxin gene PCR-end point of *Bacillus cereus* (ISO 7932:2004/AMD 1:2020) [49], enumeration of total yeast and mould (TYMC) (ISO 21527:2008) [50] and detection of *Listeria monocytogenes* (ISO 11290-1:2017) [51] and *Salmonella* (UNI EN ISO 6579-1:2017) [52].

Results were interpreted in conformity with the general requirements and guidance for microbiological examinations of food and animal feeding stuffs (ISO 7218:2007) [42], and the unit of measurement used is CFU/mL.

After isolation, bacteria were identified using API identification kits (Biomérieux, Florence) and VITEK^®^ MS through MALDI-TOF technology (Biomérieux, Florence).

To examine product sterility, microbiological stability and packaging integrity, samples were stocked in their original packages in a thermostatic chamber at 31 °C for 21 days.

Organoleptic characteristics (appearance, colour, odour, residues) were judged by two operators double blinded to the study.

## 3. Results and Discussion

### 3.1. Pesticides Analysis

Most of the samples analysed did not present detectable pesticide residue, as shown in Table 3. Fipronil sulfone, piperonyl-butoxide and pirimiphos-methyl were the residue compounds quantified in all three matrices, and all of them had very low concentrations, in particular in pasteurized soy and rice matrices, and in all the organic and without sugar beverages, no residues have been found. In all the analysed samples, only 4 soy, 4 rice and 3 oats samples presented detectable residues. Among all soy-based samples, fipronil sulfone was detected in just one fortified sample (0.12 μg/L), piperonyl-butoxide in two UHT samples (2.03 ± 1.02 μg/L; 3.18 ± 1.59 μg/L) and pirimiphos-methyl in one UHT sample (0.068 μg/L). Among all rice samples, just in three UHT samples were detected residues (piperonyl-butoxide: 2.22 ± 1.11, 6.6 ± 3.30, 3.74 ± 1.87 μg/L and pirimiphos-methyl: 1.19 ± 0.60 μg/L). In oat samples, just in two UHT samples were detected pesticide residues (pirimiphos-methyl: 6.65 ± 3.32 μg/L and piperonyl-butoxide: 7.18 ± 3.59, 23.7 ± 11.9 μg/L). All concentrations detected were very low, and samples were complied with the EU regulations. In Table 3 are summarised residue concentrations detected.

Cereal and legume concentration in milk alternatives drinks is quite low, and it can reach percentages between 5.8 and 17%, as can be proved by reading Tables S3–S5 in the Supplementary Materials. However, even residue concentrations corrected considering cereal and legumes percentage, do not exceed European LMR.

Below (Table 4) is reported the concentration values of pesticides in the three matrices investigated, calculated considering the cereal and legume percentage values reported on the packaging labels (the percentages are reported in the Supplementary Materials).

Piperonyl-butoxide is one of the most common chemical synergists [53] that is added to pyrethroids to inhibit enzyme degradation or resistance to the pesticide, thus increasing its efficiency [54]. European regulation does not include piperonyl-butoxide in the framework legislation in the field of plant protection products (EC REG. 396/2005) [55]. In Italy, piperonyl-butoxide is regulated in organic farming by EC REG. 834/2007 [56] and by EC REG. 1107/2009 [57].

In laboratory routine analyses on cereal matrices, piperonyl-butoxide is often present together with pirimiphos-methyl residues, suggesting a ready-to-use co-formulate.

Pirimiphos-methyl is an organophosphate insecticide commonly used in grain storage that inhibits the enzyme acetylcholinesterase of the nervous system, causing the consequent accumulation of the neurotransmitter acetylcholine, which is toxic for insects and for birds, amphibians and mammals [58]. The maximum residue limit is set at 0.5 mg/kg for soybeans and rice and 5 mg/kg for oats, as reported in EC REG. 396/2005 [55].

Fipronil sulfone is a metabolite of fipronil contained in commercial antiparasitic products for pets and came to public attention last year following its illegal use against the red lice in industrial egg production and laying hen farms [59,60,61,62]. Fipronil sulfone is a reversible inhibitor of the γ-aminobutyric acid receptor (GABA) [63,64] as well.

In this study, it is difficult to go back to the origin of the contamination. Residues seems to be correlated just with UHT drinks, but more trial investigation will help to understand what may be the causes. Pesticide residues were present at very low concentrations, and the presence could depend on the different raw cereals or legumes used for the drink production. Industrial process could significantly affect the residue levels of pesticides contained therein and/or thereon. Due to the physico-chemical properties of the residues, residue concentration may decrease or increase in processed fractions compared to the initial concentration in the raw agricultural commodity. The resulting ratio between processed fraction and raw agricultural ingredient is denoted as processing factor (P*f*) [65]. In the present study, no process factor was taken into account. However, future studies about milk alternatives need to consider P*f*. Processing studies are fundamental to decide on compliance of residues in processed products with legal standards for the raw agricultural commodity and to refine dietary exposure estimation of humans and livestock with respect to residues in processed products [65].

### 3.2. Microbiological Analyses

Qualitative analysis about pathogen bacteria provided negative results in all samples of soy drinks.

Two samples showed yeast and mould growth, and all the other samples recorded a load of <1 CFU/mL for each microbiological analysis. The TAMBC registered a load of <1 CFU/mL on 29 samples, 1–4 CFU/mL on one sample, 5–10 CFU/mL on one sample and >100 CFU/mL on one sample.

Qualitative analysis about pathogen bacteria provided negative results in all samples of oat drinks.

The TYMC results showed a load of 4 CFU/mL on three samples, and all the other samples recorded a load of <1 CFU/mL for each microbiological analysis. In one sample, contamination by *Bacillus cereus* was detected: a single suspect colony was isolated on MYP culture medium, and the presence of the microorganism was confirmed through phenotypic identification procedures (Table 4). The search for the cereulid-toxin gene by PCR-end point showed negative results.

The TAMBC and BPA-RPF (Baird-Parker agar with Rabbit Plasma Fibrinogen supplement) culture media recorded loads of <4 CFU/mL on one sample and two samples, respectively. Two oat drinks had recorded lactose-fermenting colonies.

Qualitative analysis about pathogen bacteria provided negative results in all samples of rice drinks.

Two samples showed a yeast and mould load of <4 CFU/mL, and all the other samples recorded a load of <1 CFU/mL for each microbiological analysis. The TAMBC, TYMC and BPA-RPF (Baird-Parker agar with Rabbit Plasma Fibrinogen supplement) culture media showed loads of <4 CFU/mL for one sample, four samples and one sample, respectively. All quantitative data are resumed in the table below, Table 5.

### 3.3. Bacterial Identification Results

Each type of plant-based milk alternative showed growth presence in different culture media, but none of the bacteria identified represent a risk for the consumers. Results of the bacterial identification are summarized in Table 6.

Microbiological results [53,54,58,59,60,61,62,63,64,65] showed that thermal processes improve microbiological safety. Pasteurization aims to reduce by a 5 log the microbial load, through the application of 62.8–65.6 °C for at least 30 min or 71.7 °C for at least 15 s, without causing major changes to the nutritional and sensory characteristics. The most popular method is ultra-high-temperature (UHT) processing, which consists of applying a higher temperature (138–145 °C) for a shorter time (1–10 s) and produces a sterile product with less organoleptic changes [66]. Despite the application of thermal processes, it has been demonstrated that, under experimental conditions, pathogens such as *Listeria monocytogenes* and *Salmonella* spp. can find fertile ground for their growth [38,39,40].

Our study did not record any positivity on the presence of food pathogens; however, one pasteurized sample of soy beverage registered a TAMBC >100 CFU/g (6700 CFU/g), showing that thermal processes cannot ensure microbiological safety or a post-treatment contamination can be possible.

## 4. Conclusions

Plant-based beverages are becoming one of the largest dairy milk alternatives on the global market [1].

Several studies showed that lactose intolerance could impair quality of life of people [27,67,68]: those individual usually reduce the intake of dairy product and also the intake of long-ripened cheese [8], which contain negligible lactose tolerable by lactose intolerant subjects [69]. This diet choice leads to a reduced calcium intake, which is linked to a higher risk of developing osteoporosis unless supplemented by the intake of calcium-fortified beverages [68]. Some hard-matured cheese such as Parmigiano Reggiano PDO and Grana Padano PDO are naturally lactose free, as they contain less than 0.01% (*w/w*) of lactose [70], which is below the Italian Health Ministry limit of 0.1% (*w/w*) [71]. Facioni et al. [70] investigated grocery shopping habits of 384 Italian lactose-intolerant subjects through a questionnaire: among those who reported not tolerating lactose-free products (20% of the total number), a large number of respondents consume plant-based beverages, while a smaller proportion consume only naturally lactose-free products such as soft, semi-hard and hard cheeses. However, without a proper diagnosis of lactose intolerance, avoiding cow’s milk and milk products is not recommended, and the consumption of oat milk and rice milk should be avoided by FPIES patients [23,24]. According to Casellas et al. [27], quality of life could be impaired by symptoms and other non-disease-related factors, such as anxiety, depression and fatigue, especially in irritable bowel syndrome patients with lactose intolerance.

Considering the large diffusion of plant-based milk alternatives among both allergic/intolerant and non-allergic consumers, it is necessary to ensure their microbiological safety. In addition, since plant-based milk and cow’s milk differ in micro and macronutrients [72], it is even more important to ensure consumers have a reliable alternative from all viewpoints, such as nutritional, chemical and microbiological ones. Concluding, it is necessary to pay attention to the substitution of cow’s milk by these alternatives considering the nutritional quality. In fact, due to the diversity of nutrient types and the amount of nutrients found in the studies analysed, it is noteworthy that most plant-based beverages cannot completely fulfil as the replacement for cow’s milk regarding nutritional quality. It should also be considered that increased consumption of plant-based beverages should go hand in hand with the development of environmentally friendly crops. Intensive soybean farming, for example, as reported by notable studies [73,74], is a leading cause of deforestation.

In this study, we showed that plant-based drinks can be a safe alternative to dairy milk according to microbiological and chemical results obtained. However, further research is needed to understand the influence of this diet, and plant-based beverages, on the gut microbiome [34,35]. Notwithstanding, proper sanitary practices are necessary, as well as a risk assessment of the production and consumption of these products with the purpose to prevent foodborne disease. Chemical investigation showed low levels of pesticide residues on plant-based milk alternatives overall. No correlation between pre-treatment and residue concentration was found. Fipronil sulfone, piperonyl-butoxide and pirimiphos-methyl residue concentrations were detected at low concentrations and in few samples. Microbiological results confirmed that thermal processes improve microbiological safety and that these products do not pose a risk for the consumer. Based on our own studies, we can conclude that consumption of plant-based beverages does not lead to pesticide exposure or microbiological risk. However, due to their recent popularity, they should be included in official control by competent authority similar to other commonly consumed food categories. Our results help to better understand microbiological and chemical hazards of milk alternative beverages and support the promotion of plant-based beverages as a valid and healthy alternative to dairy milk. Future studies should include a processing factor issue in order to support scientific background indispensable for correct decisions on residue compliance and estimates of humans’ dietary exposures.

## Figures and Tables

**Table 1:** Nutritional tables of oat, soy, rice and whole cow’s milk for 100 g of samples.

Component Name		Oat Beverages ^a^ (100 g)	Soy Beverages ^b^ (100 g)	Rice Milk ^c^ (100 g)	Whole Milk ^d^ (100 g)
Proximates	Water	90.6 g	92.4 g	89.28 g	88.1 g
	Energy (Atwater general factors)	48 kcal	38 kcal	47 kcal	61 kcal
	Nitrogen	0.13 g	0.57 g	/	0.51 g
	Protein	0.8 g	3.55 g	0.028 g	3.27 g
	Total lipid (fat)	2.75 g	2.12 g	0.97 g	3.2 g
	Ash	0.79 g	0.64 g	/	0.8 g
Carbohydrates	Carbohydrates, by difference	5.1 g	1.29 g	9.17 g	4.63 g
	Fibre	<0.75 g	<0.45 g	0.3 g	/
	Sugar, total (sucrose, glucose, fructose, lactose, maltose, galactose)	2.32 g	0.56 g	5.28 g	4.81 g
Oligosaccharides	Sum of raffinose, stachyose, verbascose	/	0.53 g	/	/
Minerals	Ca	148 mg	101 mg	118 mg	123 mg
	Fe	0.26 mg	0.54mg	0.2 mg	0
	Mg	5.9 mg	21.5 mg	11 mg	11.9 mg
	P	89 mg	69 mg	56 mg	101 mg
	K	148 mg	158 mg	27 mg	150 mg
	Na	42 mg	34 mg	39 mg	38 mg
	Zn	0.09 mg	0.31 mg	0.13 mg	0.42 mg
	Cu	0.027 mg	0.108 mg	0.037 mg	0.001 mg
	Mn	0.126 mg	0.280 mg	/	0 mg
	I	/	<0.10 µg	/	37.9 µg
	Se	2.5 µg	1.9 µg	2.2 µg	1.9 µg
	Mo	10.1 µg	58.4 µg	/	/
Vitamins	Thiamin	0.04 mg	0.063 mg	0.027 mg	0.056 mg
	Riboflavin	0.281 mg	0.084 mg	0.142 mg	0.138 mg
	Niacin	0.096 mg	0.236 mg	0.39 mg	0.105 mg
	Vitamin B-6	0.006 mg	0.055 mg	0.039 mg	0.061 mg
	Biotin	1.41 mg	3.34 µg	/	/
	Folate, total	<6 mg	20 µg	2 µg	/
	Choline, total	/	/	2.1 mg	17.8 mg
	Betaine	/	/	/	0.7 mg
	Vitamin B-12	0.51 mg	0.39 µg	0.63 µg	0.54 µg
	Vitamin A	/	58 µg	63 µg	32 µg
	Retinol	85 mg	58 µg	63 µg	31 µg
	Carotene, total	/	/	/	7 µg
	Lutein + zeaxanthin	/	8 µg	/	6 µg
	Vitamin E (α-tocopherol)	/	0.16 mg	0.47 mg	0.05 mg
	γ,δ-tocopherol	/	2.29 mg	/	/
	Vitamin D (D2+D3)	1.7 mg	0.68 µg	1 µg	0.96 µg
Amino acids	Tryptophan	0.009 mg	0.046 g	/	0.043 g
	Threonine	0.022 g	0.128 g	/	0.154 g
	Isoleucine	0.025 g	0.145 g	/	0.173 g
	Leucine	0.081 g	0.249 g	/	0.333 g
	Lysine	0.061 g	0.221 g	/	0.298 g
	Methionine	0.01 g	0.046 g	/	0.09 g
	Phenylalanine	0.072 g	0.175 g	/	0.161 g
	Tyrosine	0.041 g	0.124 g	/	0.062 g
	Valine	0.032 g	0.142 g	/	0.207 g
	Arginine	0.082 g	0.269 g	/	0.127 g
	Histidine	0.018 g	0.098 g	/	0.097 g
	Alanine	0.038 g	0.139 g	/	0.11 g
	Aspartic acid	0.082 g	0.396 g	/	0.27 g
	Glutamic acid	0.15 g	0.619 g	/	0.788 g
	Glycine	0.056 g	0.141 g	/	0.069 g
	Proline	0.072 g	13.7 g	/	0.333 g
	Serine	0.056 g	0.168 g	/	0.188 g
	Hydroxyproline	0.01 g	<0.01 g	/	0 g
	Cysteine	0.025 g	0.058 g	/	0.038 g
Isoflavonoids	Sum of daidzein, genistein, daidzin, genistin, glycitin	/	33.91 mg	/	/

^a^: https://fdc.nal.usda.gov/fdc-app.html#/food-details/2257046/nutrients; ^b^: https://fdc.nal.usda.gov/fdc-app.html#/food-details/1999630/nutrients; ^c^: https://fdc.nal.usda.gov/fdc-app.html#/food-details/1097552/nutrients; ^d^: https://fdc.nal.usda.gov/fdc-app.html#/food-details/746782/nutrients (accessed on 7 May 2023).

**Table 2:** Distribution of samples.

Plant-Based Beverages	No. of Analysed Samples	UHT	Pasteurized	Organic	Fortified *	Flavoured **	Without Sugar Added
**Soy**	33	22	11	4	3	1	3
**Rice**	17	14	3	12	0	0	4
**Oat**	10	10	0	6	0	0	5

*: vitamins added; **: chocolate flavour added.

**Table 3:** Concentration and standard deviation of fipronil sulfone, piperonyl-butoxide and pirimiphos-methyl residues detected in plant-based drinks.

Plant-Based Drinks	Treatments	Fipronil Sulfone (μg/L ± sd)	Piperonyl-Butoxide (μg/L ± sd)	Pirimiphos-Methyl (μg/L ± sd)
**Soy**	UHT	/	2.03 ± 1.02 3.18 ± 1.59	0.068 *
	Pasteurized	/	/	/
	Organic	/	/	/
	Fortified	0.12 *	/	/
	Flavoured	/	/	/
	Without sugar	/	/	/
**Rice**	UHT	/	6.6 ± 3.30 2.22 ± 1.11 3.74 ± 1.87	1.19 ± 0.60
	Pasteurized	/	/	/
	Organic	/	/	/
	Without sugar	/	/	/
**Oat**	UHT	/	7.18 ± 3.59 23.7 ± 11.9	6.65 ± 3.32
	Organic	/	/	/
	Without sugar	/	/	/

The concentrations marked with an asterisk are estimates; for these analyses, the identification criteria were verified but the value was outside the concentration range for the method that had been tested. However, NRL-CF considered the estimated value useful to express the order of magnitude of the pesticide contamination. Since this is an estimate, it is not possible to express the relative standard deviation.

**Table 4:** Concentration and standard deviation of fipronil sulfone, piperonyl-butoxide and pirimiphos-methyl residues detected in plant-based drinks calculated considering cereal and legume percentage.

Plant-Based Drinks	Treatments	Fipronil Sulfone (μg/L ± sd)	Piperonyl-Butoxide (μg/L ± sd)	Pirimiphos-Methyl (μg/L ± sd)
**Soy**	UHT	/	32.22±16.19 46.76±23.38	0.97 *
	Pasteurized	/	/	/
	Organic	/	/	/
	Fortified	1.88 *	/	/
	Flavoured	/	/	/
	Without sugar	/	/	/
**Rice**	UHT	/	13.06 ± 6.53 38.82 ± 19.41 22 ± 11	7 ± 3.53
	Pasteurized	/	/	/
	Organic	/	/	/
	Without sugar	/	/	/
**Oat**	UHT	/	89.75 ± 44.88 237 ± 119	66.5 ± 32.2
	Organic	/	/	/
	Without sugar	/		/

The concentrations marked with an asterisk are estimates; for these analyses, the identification criteria were verified but the value was outside the concentration range for the method that had been tested. However, NRL-CF considered the estimated value useful to express the order of magnitude of the pesticide contamination. Since this is an estimate, it is not possible to express the relative standard deviation.

**Table 5:** Microbiological results: TAMBC (total aerobic mesophilic bacteria count); CPS (coagulase-positive staphylococci); SRAB (sulphite-reducing anaerobic bacteria); TYMC (total yeast and mould).

Matrices	Quantitative Analysis						
	TAMBC	CPS	Total Coliforms	*Enterobacteriaceae*	SRAB	*B. cereus*	TYMC
	(UFC/mL)						
**Soy**	<1	<1	<1	<1	<1	<1	<1
	<1	<1	<1	<1	<1	<1	<1
	<1	<1	<1	<1	<1	<1	19
	<1	<1	<1	<1	<1	<1	<1
	<1	<1	<1	<1	<1	<1	<1
	<1	<1	<1	<1	<1	<1	<1
	<1	<1	<1	<1	<1	<1	<1
	<1	<1	<1	<1	<1	<1	<1
	<1	<1	<1	<1	<1	<1	<1
	<1	<1	<1	<1	<1	<1	<1
	<1	<1	<1	<1	<1	<1	<1
	<1	<1	<1	<1	<1	<1	<1
	<1	<1	<1	<1	<1	<1	<1
	<1	<1	<1	<1	<1	<1	<1
	<1	<1	<1	<1	<1	<1	<1
	<1	<1	<1	<1	<1	<1	<1
	<1	<1	<1	<1	<1	<1	<1
	<1	<1	<1	<1	<1	<1	<1
	<1	<1	<1	<1	<1	<1	<1
	<1	<1	<1	<1	<1	<1	<1
	<1	<1	<1	<1	<1	<1	<1
	<1	<1	<1	<1	<1	<1	<4
	<1	<1	<1	<1	<1	<1	<1
	5	<1	<1	<1	<1	<1	<1
	<1	<1	<1	<1	<1	<1	<1
	<1	<1	<1	<1	<1	<1	<1
	<1	<1	<1	<1	<1	<1	<1
	15	<1	<1	<1	<1	<1	<1
	6700	<1	<1	<1	<1	<1	<1
	<1	<1	<1	<1	<1	<1	<1
	<4	<1	<1	<1	<1	<1	<1
	<1	<1	<1	<1	<1	<1	<1
	<1	<1	<1	<1	<1	<1	<1
**Oats**	<1	<1	<1	<1	<1	<1	<1
	<1	<1	<1	<1	<1	<1	4
	<1	<4	<1	<1	<1	<4	<4
	<1	<1	<1	<1	<1	<1	<1
	<1	<1	<1	<1	<1	<1	4
	<1	<1	<1	<1	<1	<1	<1
	<4	<1	<1	<1	<1	<1	<1
	<1	<1	<1	<1	<1	<1	<1
	<1	<4	<1	<1	<1	<1	<1
	<1	<1	<1	<1	<1	<1	<1
**Rice**	<1	<1	<1	<1	<1	<1	<1
	<1	<1	<1	<1	<1	<1	<1
	<1	<1	<1	<1	<1	<1	<4
	<1	<1	<1	<1	<1	<1	<1
	<1	<1	<1	<1	<1	<1	<1
	<1	<1	<1	<1	<1	<1	<4
	<1	<1	<1	<1	<1	<1	<1
	<1	<1	<1	<1	<1	<1	<1
	<1	<1	<1	<1	<1	<1	<1
	<1	<1	<1	<1	<1	<1	<4
	<1	<1	<1	<1	<1	<1	<1
	<1	<1	<1	<1	<1	<1	<1
	<1	<1	<1	<1	<1	<1	<1
	<1	<1	<1	<1	<1	<1	<4
	<4	<1	<1	<1	<1	<1	<1
	<1	<4	<1	<1	<1	<1	<1
	<1	<1	<1	<1	<1	<1	<1

**Table 6:** Bacterial identification in different types of plant-based drinks.

Plant-Based Drinks	Identification Result	Identification Method
**Soy**	*Leifsonia aquatica*	API CORYNE V4.0
	*Bacillus firmus*	API 50 CHB V4.1
	*Bacillus simplex*	VITEK^®^ MS
	*Bacillus simplex*	VITEK^®^ MS
	*Bacillus firmus*	API 50 CHB V4.1
	*Staphylococcus warneri*	VITEK^®^ MS
	*Staphylococcus warneri*	VITEK^®^ MS
**Oat**	*Staphylococcus warneri*	VITEK^®^ MS
	*Staphylococcus warneri*	VITEK^®^ MS
**Rice**	*Micobacterium oxydans*	VITEK^®^ MS
	*Finegoldia magna*	VITEK^®^ MS
	*Micrococcus luteus*	VITEK^®^ MS
	*Staphylococcus epidermidis*	VITEK^®^ MS
	*Bacillus subtilis*	VITEK^®^ MS
	*Bacillus* spp. *(B. amyloliquefaciens/B. vallismortis)*	VITEK^®^ MS
	*Staphylococcus warneri*	VITEK^®^ MS

## Data Availability

No new data were created or analyzed in this study. Data sharing is not applicable to this article.

## Supplementary Materials

The following supporting information can be downloaded at: https://www.mdpi.com/article/10.3390/nu15102258/s1, Table S1: Pesticides transition. Pesticides name, parent ion (*m*/*z*), quantitative and qualitative ion (*m*/*z*), collision energy CE (V); Table S2: Validated pesticides LOQs; Table S3: Declared soy percentage (%) in soy drink; Table S4: Declared rice percentage (%) in rice drink; Table S5: Declared oat percentage (%) in oat drink.
