# Supplementary material for: Soy, Rice and Oat Drinks: Investigating Chemical and Biological Safety in Plant-Based Milk Alternatives
Source: Nutrients. 2023 May 10;15(10):2258. doi: 10.3390/nu15102258 (PMC10221834; doi:10.3390/nu15102258)
Supplement: Supplementary file 1 [file nutrients-15-02258-s001.zip › nutrients-2371905-supplementary.pdf]

Supplementary Material

Table S1. Pesticides transition. Pesticides name, parent ion (m/z), quantitative and qualitative ion (m/z), collision energy CE (V).

Pesticide	Parent Ion (m/z)	Quantitation and Qualification Ions (m/z)	Collision Energy (V)
Dichlorvos	185	93.1	10
	109	79	5
Biphenyl	154.1	152.1	25
	154.1	153.1	10
Methacrifos	125	47.1	10
	208	180.1	5
2-Phenylphenol	170.1	169.1	10
	169.1	141.1	10
Diphenylamine	168.2	167.1	15
	169.2	168.2	10
Chlorpropham	213.1	127.1	10
	213.1	171.1	5
Trifluralin	306.1	264.1	5
	306.1	206.1	10
Cadusafos	159	97	15
	159	131	5
Phosmet	160.1	77.1	20
	160.1	51.1	35
α-HCH	181	145	15
	218.9	183	5
HCB	283.8	248.9	15
	283.8	213.9	30
Aldrin	262.8	193	30
	262.8	191	30
Fenthion	278	109.1	15
	278	169.1	15
Parathion	291.1	109.1	10
	235	139.1	5
Triadimefon	208	181.1	5
	208	127.1	15
Isocarbophos	230	212.1	10
	109.9	64	10
Pirimiphos	304.1	168.1	10
	318.1	166.2	10
Cyprodinil	224.1	208.2	15
	225.2	210.2	15
Pendimethalin	252.1	162.2	10
	252.1	161.2	15
Chlorfenvinphos	266.9	159.1	15
	323	267	10
Oxychlordane	386.9	263	10
	386.9	287	15

Heptachlor-exo-epoxide	352.8	262.9	10
	354.8	264.9	10
Phosmet-oxon	160	133.1	10
	160	77.1	20
p,p′-DDT	235	165.1	20
	237	165.1	20
Propargite	173.1	135.1	15
	201.1	81.1	10
TPP (SI)	325	169.1	15
	326.1	233.1	10
Piperonyl-butoxide	176.1	103.1	10
	176.1	131.1	20
Resmethrin	171.1	128.1	15
	171.1	143.1	5
Spiromesifen	272.1	209.2	10
	272.1	254.2	5
Endrin-ketone	316.9	281	5
	245	173.1	25
Iprodione	314.1	245.1	10
	316.1	247.1	10
Tetramethrin	164.1	107.1	10
	164.1	77.1	25
Bifenthrin	181.1	166.2	10
	181.1	165.1	25
Esfenvalerate	125	89.1	20
	167.1	125.1	10
Difenoconazole	323	265.1	10
	265	139.1	35
Desmedipham	181.1	109.1	10
	109.1	8.1	10
β-HCH	181	145	15
	218.9	183	5
Lindane (γ-HCH)	181	145	15
	218.9	183	5
Diazinon	137.1	84.1	10
	152.1	137.1	5
Pyrimethanil	198.1	118.1	30
	198.1	182.1	20
Chlorothalonil	265.8	133	35
	263.9	133	35
Paraoxon-methyl	230	106.1	15
	230	136.1	5
Tefluthrin	177	127.1	15
	177	137.1	15
Chlorpyrifos-Methyl	286,0	93.1	20
	286	271	10
Malaoxon	127	99.1	5
	127	109.1	10
Metribuzin	198.1	82.1	15
	198.1	110.1	10

Fipronil	366.9	213	25
	369	215	25
Heptachlor-endo-epoxide	217	182	15
	183	155	15
Phenthoate	274	121.1	10
	246	121.1	5
Procymidone	283	96.1	10
	285	96.1	10
Triadimenol	128	65.1	20
	168.1	70.1	10
trans-Chlordane	372.8	266	20
	374.8	266	20
α-Endosulfan	241	206	10
	207	172.1	10
cis-Chlordane	372.8	266	20
	374.8	266	20
Mepanipyrim	222.2	206.2	25
	222.2	118.2	35
Fludioxonil	154.1	127.1	25
	248	127.1	10
Isoprothiolane	162	85	15
	204.1	118.1	5
EPN	169	77.1	20
	169	141.1	5
Bromopropylate	340.9	185	15
	343	185	15
Bromuconazole	294.9	173	10
	173	109	25
Methoxychlor	227.1	169.1	25
	227.1	141.1	30
Fenpropathrin	265.1	89.1	10
	265.1	210.2	30
Tebufenpyrad	276.1	171.1	10
	333.2	171.1	15
Fenazaquin	160.2	117.1	20
	145.1	91.1	25
Phosalone	182	75.1	30
	182	111.1	15
λ-Cyhalothrin	181.1	152.1	20
	197	141.1	10
Fenarimol	139	75.1	25
	139	111.1	15
Acrinathrin	208.1	181.1	5
	181.1	152.1	20
Deltamethrin	252.9	172.1	5
	252.9	93.1	15
Azoxystrobin	344.1	329.2	10
	388.1	360.2	10
Vinclozolin	198	145	15
	187	124	15
Parathion-methyl	263	109.1	10

	263	79.1	25
Tolclofos-methyl	265	250	10
	265	220	20
Metalaxyl	206.1	132.1	15
	160.1	130.1	15
Heptachlor	100	65.1	10
	271.8	236.9	10
Pirimiphos-methyl	290.1	125.1	20
	305.1	180.2	5
Fenitrothion	277	260.1	5
	277	109.1	15
Malathion	173.1	127.1	5
	173.1	99.1	10
Metolachlor	238.1	162.2	10
	162.1	132.1	20
Chlorpyrifos	314	258	10
	316	260	10
Fenpropimorph	128.1	70.1	10
	303.3	128.2	5
Profenofos	339	269	10
	337	267	10
p,p′-DDE	246	176.1	30
	317.9	248.1	15
Dieldrin	276.9	241	5
	276.9	207.1	20
Fipronil-sulfone	383	255.1	15
	385	257	15
Kresoxim-methyl	206.1	116.1	5
	116.1	89.1	15
Endrin	279	243.1	10
	281	245	10
β- Endosulfan	241	206	10
	207	172.1	10
Ethion	231	129	20
	153	97	10
Oxadixyl	163.1	132.1	10
	163.1	117.1	20
p,p′-DDD + o,p′-DDT	235	165.1	20
	237	165.1	20
Endosulfan-sulfate	271.8	236.9	10
	238.8	204	10
Fluquinconazole	340.1	298.1	10
	340.1	108.1	35
Permethrin	183.1	168.1	10
	183.1	153.1	10
Pyridaben	147.1	117.1	20
	147.1	132.2	10
Cyfluthrin	226.1	206.1	10
	206.1	151.2	15
Boscalid	140	112	10
	140	76.1	20

Cypermethrin	163	127.1	5
	163	91.1	10
Bixafen	413	159.1	10
	159	139.1	10
Flucythrinate	157.1	107.1	10
	199.1	157.1	5
Etofenprox	163.1	135.1	10
	163.1	107.1	15
Fenvalerate	125	89.1	20
	167.1	125.1	10
τ-Fluvalinate	250.1	200.1	15
	250.1	55.1	15

Table S2: Validated pesticides LOQs.

Analita	LOQ	Analita	LOQ
Acrinathrin	0.002	Fluquinconazole	0.002
Azoxystrobin	0.002	Heptachlor-exo-epoxide	0.005
Bifenthrin	0.002	Iprodione	0.002
Bixafen	0.002	Isocarbophos	0.002
Boscalid	0.002	Isoprothiolane	0.002
Bromopropylate	0.002	Kresoxim-methyl	0.002
Bromuconazole	0.002	ʎ-Cyhalothrin	0.002
Chlorfenvinphos	0.002	Malaoxon	0.005
Chlorpyrifos	0.005	Malathion	0.005
cis-Chlordane	0.005	Mepanipyrim	0.005
Cyfluthrin	0.002	Metalaxyl	0.005
Cypermethrin	0.002	Methoxychlor	0.002
Cyprodinil	0.005	Metolachlor	0.005
p,p′-DDD + o,p′-DDT	0.004	Metribuzin	0.005
p,p′-DDE	0.002	Oxadixyl	0.002
p,p′-DDT	0.002	Paraoxon-methyl	0.005
Deltamethrin	0.002	Parathion	0.002
Dieldrin	0.005	Pendimenthalin	0.005
Difenoconazole	0.002	Permethrin	0.005
α-Endosulfan	0.005	Phenthoate	0.002
β- Endosulfan	0.005	Phosalone	0.002
Endosulfan-sulfate	0.002	Phosmet	0.002
Endrin	0.005	Phosmet-oxon	0.002
Endrin-ketone	0.005	Piperonil-butoxide	0.002
EPN	0.002	Pirimiphos	0.005
Esfenvalerate	0.002	Pirimiphos-methyl	0.005
Ethion	0.002	Procymidone	0.002

Etofenprox	0.002	Profenofos	0.002
Fenarimol	0.002	Propargite	0.005
Fenazaquin	0.005	Pyridaben	0.005
Fenitrothion	0.005	Resmethrin	0.002
Fenpropathrin	0.002	Spiromesifen	0.002
Fenpropimorph	0.005	τ-Fluvalinate	0.002
Fenthion	0.005	Tebufenpyrad	0.002
Fenvalerate	0.002	Tetramethrin	0.002
Fipronil	0.002	trans-Chlordane	0.005
Fipronil-sulfone	0.002	Triadimefon	0.002
Flucythrinate	0.002	Vinclozolin	0.005
Fludioxonil	0.002	β-HCH	0.005

Table S3: Declared soy percentage (%) in soy drink.

n°	Declared soy (%)
1	6.4
2	6
3	6.4
4	8.7
5	6.3
6	7
7	6.4
8	7.5
9	6
10	6.8
11	6.8
12	5.9
13	7.5
14	8
15	7.2
16	6.7
17	8
18	7.5
19	6.9
20	7.2
21	7.2
22	7.2
23	5.8
24	6.8
25	6.4
26	6.4
27	8
28	6.4
29	6.4
30	6.4
31	6.4
32	6.8
33	8

Table S4: Declared rice percentage (%) in rice drink.

n°	Declared rice (%)
1	17
2	17
3	16
4	12
5	17
6	17
7	17
8	17
9	17
10	17
11	17
12	17
13	16
14	16
15	17
16	17
17	17

Table S5: Declared oat percentage (%) in oat drink.

n°	Declared oat (%)
1	8
2	11
3	10
4	16
5	14
6	11
7	14
8	10
9	12
10	12
